# The impact of socio-economic status on health related quality of life for children and adolescents with heart disease

**DOI:** 10.1186/1477-7525-11-99

**Published:** 2013-06-18

**Authors:** Amy Cassedy, Dennis Drotar, Richard Ittenbach, Shawna Hottinger, Jo Wray, Gil Wernovsky, Jane W Newburger, Lynn Mahony, Kathleen Mussatto, Mitchell I Cohen, Bradley S Marino

**Affiliations:** 1 Department of Pediatrics, Division of Biostatistics and Epidemiology, Cincinnati Children’s Hospital Medical Center, University of Cincinnati College of Medicine, 3333 Burnet Avenue, Cincinnati, OH, 45229, USA; 2 Department of Sociology, McMicken College of Arts and Sciences, University of Cincinnati, Cincinnati, USA; 3 Department of Pediatrics, Division of Behavioral Medicine and Clinical Psychology, Cincinnati Children’s Hospital Medical Center, University of Cincinnati College of Medicine, Cincinnati, OH, USA; 4 Department of Pediatrics, Divisions of Allergy and Immunology, Cincinnati Children’s Hospital Medical Center, University of Cincinnati College of Medicine, Cincinnati, OH, USA; 5 Cardiorespiratory Division, Great Ormond Street Hospital for Children NHS Foundation Trust, London, UK; 6 Department of Anesthesiology and Critical Care, Division of Cardiology, The Children’s Hospital of Philadelphia, Philadelphia, PA, USA; 7 Division of Critical Care Medicine, The Children’s Hospital of Philadelphia, Philadelphia, PA, USA; 8 Department of Cardiology, Children's Hospital Boston, Boston, MA, USA; 9 Department of Pediatrics, Harvard Medical School, Boston, MA, USA; 10 Department of Pediatrics, Division of Cardiology, University of Texas Southwestern Medical Center at Dallas, Dallas, TX, USA; 11 Department of Pediatrics, Division of Cardiology, Children‘s Hospital of Wisconsin, Milwaukee, WI, USA; 12 Department of Pediatrics, Division of Cardiology, Phoenix Children‘s Hospital, Phoenix, AZ, USA; 13 Department of Pediatrics, Divisions of Cardiology and Critical Care Medicine, Cincinnati Children’s Hospital Medical Center, University of Cincinnati College of Medicine, Cincinnati, OH, USA

## Abstract

**Background:**

Socioeconomic status (SES) is known to influence children’s health-related quality of life. Many SES indicators assess distinct dimensions of a family’s position rather than measuring the same underlying construct. Many researchers, however, see SES indicators as interchangeable. The primary aim of this study was to determine which measure of SES had the strongest impact on health-related quality of life.

**Methods:**

This is a secondary analysis of the Pediatric Cardiac Quality of Life Inventory Validation Study. The SES variables were family income, Hollingshead Index (occupational prestige), and highest parent educational attainment level. Health-related quality of life was measured using the Pediatric Cardiac Quality of Life Inventory. Correlations tested the relationship among the three SES indicators. Regression-based modeling was used to calculate the strength of the association between SES measures and the Pediatric Cardiac Quality of Life Inventory.

**Results:**

The correlations among the SES measures were moderately high, with the correlation between the Hollingshead Index and parental education being r = 0.62 (95% CI = 0.56-0.65). There were equally high correlations between family income and the Hollingshead (r = 0.61, 95% CI = 0.57-0.65) and a slightly lower correlation between family income and parental education (r = 0.55, 95% CI = 0.52-0.59). Family income had the highest explanatory value compared to the Hollingshead Index or parental educational attainment, while controlling for sex, race, current cardiac status, and original diagnosis, accounting for 4-5% of the variation in patient and parent Pediatric Cardiac Quality of Life Inventory Total score, respectively, compared to the other SES measures.

**Conclusion:**

Family income as an SES measure demonstrated the greatest fidelity with respect to health-related quality of life as measured by the Pediatric Cardiac Quality of Life Inventory across respondent groups and explained more of the variation compared to the Hollingshead Index or highest parental educational attainment.

## Introduction

Family income, occupational prestige, and educational attainment are measures of Socio-Economic Status (SES) that have been found to influence an individual’s life opportunities. Life opportunities can manifest themselves in various ways, such as availability of resources to an individual within the health care system or an individual’s perception about their Health Related Quality of Life (HRQOL), defined as the impact of a specific illness, injury, medical treatment, or health services policy on quality of life [1]. The relationship between higher levels of SES and higher scores on HRQOL in children has been well established [2-4]. Recent studies have also described the relationship between the specific effects of pediatric chronic disease in children and HRQOL. Children and adolescents with chronic disease will often have a lower HRQOL as seen in Duchenne Muscular Dystrophy [5], asthma [6], and heart disease [7-9].

Structural inequality due to socio-demographic, cultural, and economic differences is often consolidated under the all-encompassing label “socio-economic status”. This inclusive categorization is based upon the assumptions that social structure is stratified, that each individual can be ranked with respect to the position they occupy in the social structure, and that each of the SES elements (demographic, cultural, economic) contributes to an individual’s societal status in this stratified system. Unequal access to resources is inherent to an individual’s status within society and can adversely affect an individual’s life opportunities [10]. There are a variety of SES indicators that often assess distinct dimensions of SES rather than measuring the same underlying construct [11]. Access to resources, skills capability and social relationships, and knowledge about health related issues all impact HRQOL. For instance, income differentials may determine access to resources [12] and can be seen as a dimension of “financial capital” [13,14], whereas occupational prestige is viewed as measuring skill capability and social relationships for the individual [12]. Educational attainment reflects knowledge about health-related behaviors [12], contributes to an individual’s future earnings [15] and can provide “human capital” such as skills and aspirations necessary for the success of the family unit [13,14]. Another dimension often overlooked by health researchers is “social capital” that provide social networks connecting individuals to the larger community [13,14].

Unfortunately, many investigators find it difficult to ascertain which SES measures are valid, which can be applied to multiple outcomes, and which measure is the most effective with a specific condition. In addition, many researchers know they must control for the affect of SES on their outcomes but see SES measures as interchangeable. Other investigators are uncomfortable asking questions about family income, relying instead on the Hollingshead Index, which many social science researchers find to be outdated and not useful [11]. Therefore, health care researchers would be best served by knowing the impact of each SES factor on health-related outcomes so that the true variation due to SES is not missed by employing a weaker SES measure. Given that critical health-related outcomes such as HRQOL are known to be sensitive to SES influences coupled with the fact that congenital heart disease (CHD) is an exemplar chronic condition, results from a study that addresses these two elements together can potentially inform researchers about SES measures that can be generalizable to the pediatric population that is experiencing chronic health conditions.

The primary aim of this study was to determine which measure of SES [family income, Hollingshead Index (occupational prestige), and highest parent educational attainment level] had the strongest impact on HRQOL score using the Pediatric Cardiac Quality of Life Inventory (PCQLI), a disease-specific HRQOL instrument for children and adolescents with congenital or acquired heart disease, after adjusting for patient gender, race, and current cardiac status. Since this study had all three SES indicators, a thorough test of each measure could be conducted.

## Methods

### Study and inventory design

This is a secondary analysis of the multi-center PCQLI Validation Study [8,16]. The PCQLI Validation Study sample was recruited from seven large pediatric cardiac centers in the United States and consisted of 1,605 patients with congenital or acquired heart disease and their parents/guardians. The study populations for this analysis consisted of 1,383 of these patients and parents/guardians pairs with valid PCQLI Total score results (86% of the entire sample) that had complete data on all three SES indicators. During the design stage of the validation study, power and sample size estimates were computed. Because there was no attempt at limiting the number of participants as long as they met the inclusionary criteria, the validation study included proportionally more upper income participants then what would be expected from the general US population. The primary PCQLI study was approved by each center’s Institutional Review Board, and written informed consent was obtained for all participants.

### Measures

#### PCQLI total score (outcome variable)

The PCQLI is an age-specific, self-administered instrument measuring perceptions of physical and psychosocial HRQOL for children and adolescents with congenital or acquired HD [7]. There are separate versions of the questionnaire for patients and parents/guardians of patients 8 to 12 years of age (Child Form, Parent of Child Form) and for patients and parents/guardians of patients 13 to 18 years of age (Adolescent Form, Parent of Adolescent Form) [7]. The instrument was developed at The Children’s Hospital of Philadelphia using focus group methodology and was further validated using samples from Massachusetts, Wisconsin, Ohio, Texas, California, and Arizona [7,8,16]. The PCQLI has been shown to be reliable, valid, and generalizable [8,16]. Test-retest reliability correlations are excellent (r = 0.78-0.90) [8]. Construct validity was substantiated by: significant associations between lower PCQLI scores and greater CHD disease severity, as well as increased number of cardiac surgeries, hospital admissions, and doctor visits; moderate to good significant correlations between patient and parent PCQLI scores (r = 0.41-0.61); and moderate to good significant correlations of PCQLI Total scores with PedsQL Total score (r = 0.70-0.76), Self-Perception Profile for Children and Adolescent Global Self-Worth (r = 0.43-0.46), Youth Self Report/Child Behavior Checklist Total Competency (r = 0.28-0.37), and Syndrome and Diagnostic and Statistical Manual of Medical Disorders-IV Oriented Scale scores (r = −0.58 to −0.30) [8]. The PCQLI was shown to be externally valid (generalizable) in a geographically diverse population: subscale to subscale correlations (0.79 for both the developmental site and the composite sites), subscale to total scale correlations (Development sample, Disease Impact 0.95, Psychosocial Impact 0.95; Composite sample, Disease Impact 0.95, Psychosocial Impact 0.94), and internal consistency (Development sample, Total Score 0.93, Disease Impact 0.90, Psychosocial Impact 0.84; Composite sample, Total Score 0.93, Disease Impact 0.89, Psychosocial Impact 0.85) were high in both samples [16].

#### Socio-economic indicators: family income, Hollingshead index, parental education (predictors)

For the original PCQLI study, parents (or parent proxies) of patients were asked questions related to SES so that the patient/parent pair had the same SES classifications. To have a thorough analysis of SES for this study, we selected three socio-economic indicators available from the PCQLI family background questionnaire: family income, Hollingshead Index (occupational prestige), and highest parent educational attainment level. Parents were asked to check the category that best described their combined household yearly income: Less than $26,000; $26,000 to $50,000; $51,000 to $75,000; $76,000 to $100,000; $101,000 to $150,000; and Greater Than $150,000. This question represented the family income variable. Occupational prestige was measured using the Hollingshead scale. The Hollingshead scale is based upon an the work of A. A. Hollingshead in which SES is a combination of an adult’s marital status, education, and occupation [17]. Even though the Hollingshead Index lacks validity and practicality according to experts in social stratification [11], it is widely used and hence serves as a benchmark of comparison with other indicators of SES. For example Limbers [18] and Marino [8] use the Hollingshead Index as a proxy for SES. We used the following Hollingshead Index categories for our analysis: Lower Status, Lower-middle Status, Middle Status, Upper-middle Status, and Upper Status. In the original study design [7], highest parent educational attainment level was a seven-category variable with the following categories: Kindergarten through 6th grade, 7th – 9th grade, 10th – 11th grade, High School Graduate, Partial College or Trade School, College Graduate, and Post-Graduate Degrees. However, there were very few respondents having less than a high school degree (less than 4% of the sample). The 4 lowest educational categories were combined to create a category “Less Than High School Degree or High School Degree Only”. The other parental educational levels remained the same: “Partial College or Trade School”, “College Graduate”, and “Post Graduate Degree”. While having more categories for this variable would be desirable, having so few cases in some cells necessitated combining categories.

#### Patient factors: race, sex, and current cardiac status (control variables)

The patient factors of age, race, gender, and current cardiac status were selected as control variables due to their potential influence on SES and/or HRQOL. Due to low numbers in many of the racial/ethnic groupings in the PCQLI Validation Study, this variable was recoded into two race categories: Caucasian and Non-Caucasian. The authors were concerned that a large number of patients in the sample were likely to have complex heart disease, potentially leading to lower HRQOL scores and masking the SES effect. In addition, the authors recognized the relationship between maternal socioeconomic status and the potential for birth defect causing a more severe type of congenital heart disease [19]. Therefore, in our study, we not only controlled for current cardiac status but original cardiac diagnosis categories with categorical variables. Current cardiac status was coded as follows: 1. structurally normal heart; 2. Unrepaired CHD (those who had CHD but did not undergo surgical or catheter-based intervention); 3. Status post (S/p) heart surgery or catheter-based intervention, or pacemaker procedure; 4. S/p transplant (as the reference category). Original cardiac diagnosis was coded as follows: 1. Two-ventricle CHD without aortic arch obstruction; 2. Two-ventricle CHD with aortic arch obstruction; 3. Single-ventricle CHD without aortic arch obstruction; 4. Single-ventricle CHD with aortic arch obstruction; 5. Non-congenital acquired heart disease (as the reference category).

### Statistical analysis

Summary statistics were calculated for all predictor [family income, Hollingshead Index (occupational prestige), parent educational attainment level] and control variables (patient race, gender, current cardiac status, and original cardiac diagnosis) to examine the distribution of the data with the outcome variable (PCQLI Total score). We also tested for collinearity between the predictor SES variables and the demographic and clinical covariates in the model, using correlation coefficients, and among the predictor variables using both correlation coefficients and tolerance tests. Since a correlation coefficient of greater than 0.60 implies a moderate relationship between variables [20], 0.60 was used as an indicator of the potential for multicollinearity. Due to non-normal distributions and in order to test for significance, the data were transformed using the square-root transformation to normalize the distribution. Means and standard deviations for non-transformed data were also presented. Predictor variables and covariates were categorical or ordinal in nature, necessitating polychoric correlations to estimate relationships among the predictors and covariates.

To test the complete model, general linear modeling was used. Socio-economic indicators were placed into their own, separate models and tested for statistical significance: PCQLI Total score as a function of the socio-economic indicator [family income, Hollingshead Index (occupational prestige), highest parent educational attainment level] after controlling for patient age, race, gender, and current cardiac status and original diagnosis. For this study, Type I analysis was used. Predictor(s) were placed into the model after all of the control variables. The partial eta-squared (η^2^) as well as the partial omega-squared (ω^2^) were used to estimate effect size [21] as well as report the amount of variation explained, after controlling for covariates. The F-statistic was used to test if the individual SES indicator was significantly related to the outcome controlling for covariates. If two predictor variables had a correlation of less than 0.60, an additional regression model with both predictors as well as covariates was created. A change in R^2^ was computed and an F-ratio test was used to determine if both SES indicators added a significant amount of variance explained to the model. A *p*-value < 0.05 was considered statistically significant for both the predictor model and categorical levels of analysis. All analyses were conducted using SAS V 9.2©.

## Results

### Population demographics and patient clinical characteristics

The study population consisted of 1,383 patients (695 Child, 688 Adolescent). Patient respondents had a mean age of 12.5 ± 3.1 years while the parent respondents had a mean age of 43.0 ± 6.3 years. Patient and parent population demographics and patient clinical characteristics are shown in Table 1. Only 7% of respondents did not answer the question about family income, which is much lower than what many social science researchers routinely anticipate on the income variable [22]. However, results in Table 2 show that the upper strata of all three SES measures have a greater number of respondents than what is expected nationally. The median family income for the United States is approximately $50,000 per year [23], but for our sample only 23.4% had a family income of less than or equal to $50,000 per year. The mean score for patient PCQLI was 75.8 ± 16.2, while the mean score for parent PCQLI was 76.3 ± 17.1.

**Table 1:** Frequency distribution of demographic and clinical variables

	**n**	**%**
	1383	100
**Patient gender**		
Male	765	55.3
Female	618	44.7
**Patient race**		
Non-Caucasian	195	14.1
Caucasian	1188	85.9
**Patient age**		
Mean, Standard Deviation	12.5	3.1
**Patient current cardiac status**		
Structurally normal	214	15.5
Unrepaired CHD	161	11.6
S/p Heart surgery or catheter-based Intervention	959	69.3
S/p Transplant	49	3.5
**Patient original diagnostic category**		
2-ventricle CHD without Aortic Arch Obstruction	664	48.0
2-ventricle CHD with Aortic Arch Obstruction	137	9.9
1-ventricle CHD Aortic Arch Obstruction	100	7.2
1-ventricle CHD with Aortic Arch Obstruction	89	6.4
HD with a structurally normal heart	393	28.4
**Parent gender**		
Male	212	15.3
Female	1171	84.7
**Parent race**		
Non-Caucasian	157	11.5
Caucasian	1212	88.5
**Parent age**		
Mean, Standard Deviation	43.0	6.3

**Table 2:** Frequency distribution of socio-economic indicators

	**n**	**%**
	1383	100
**Family income**		
Less than $26,000	106	7.7
$26-50,000	217	15.7
$51-75,000	255	18.4
$76-100,000	286	20.7
$101-150,000	285	20.6
Greater than $150,000	234	16.9
**Hollingshead index**		
Lower status	52	3.8
Lower-middle status	89	6.4
Middle status	229	16.6
Upper-middle status	615	44.5
Upper status	398	28.8
**Highest parent educational attainment level**		
Less than High School Degree or High School Degree only	301	21.8
Partial College or Trade School	365	26.4
College Graduate	507	36.7
Post Graduate Degree	210	15.2

When testing for collinearity, with the exception of patient’s race, the correlations between the predictor variables (family income, Hollingshead Index, and parental educational attainment level) and all other covariates (demographic and clinical) were extremely low ranging, from r = 0.0008 to 0.05. The correlation between patient’s race and family income was moderate (r = 0.41), while the correlation between patient’s race and parental education (r = 0.25) and Hollingshead Index (r = 0.29) were both low. Not surprisingly, the correlations among the SES measures were moderately high, with the correlation between the Hollingshead Index and parental educational attainment level being r = 0.62 as well as between family income and the Hollingshead (r = 0.61) indicating the potential for multicollinearity, thus the Hollingshead Index was not entered into a model with the other predictors. A slightly lower correlation between family income and parental educational attainment level (r = 0.55) was observed.

### Impact of family income, Hollingshead index (occupational prestige), and parental educational attainment level on HRQOL score

Individually, family income had the highest explanatory value compared to the Hollingshead Index or parental educational attainment level, while controlling for all covariates in the model for both patient and parent responses (Table 3). Since parental educational attainment level and family income were moderately correlated, both SES indicators were added to the parent model to determine whether each provides a unique and significant contribution to the portion of the variance explained. The amount of variance explained for this model was 17.4%. There was no multicollinearity with both SES indicators in the model (Tolerance statistic for parental educational attainment level = 0.72 and Tolerance statistic for family income = 0.68), thus no further inquiries about collinearity were deemed necessary. However, when the two predictors were in the model, the relative contribution of parental educational attainment level was not significant (*F* Value = 5.08), whereas family income remained significantly related to parent-reported PCQLI score (*F* Value = 12.13).

**Table 3:** Percent Variance Explained for Patient and Parent PCQLI Total score by SES Indicators Controlling for Demographic and Clinical Variables

**Patient Model**							
**Source of variation**	**Sum of square**	**df**	**Mean square**	**F value**	**Pr > F**	**Partial** **η**^**2**^	**Partial** **ω**^**2**^
*Covariates: Patient characteristics*							
Patient sex	47.4	1	47.4	17.9	<0.0001	0.011	0.011
Patient race	78.7	1	78.7	29.8	<0.0001	0.019	0.018
Patient age	159.6	1	159.6	60.4	<0.0001	0.038	0.038
Current cardiac status of patient	125.4	3	41.8	15.8	<0.0001	0.030	0.028
Original cardiac diagnosis of patient	107.8	4	26.9	10.2	<0.0001	0.026	0.023
*Model 1:*							
Hollingshead index	52.6	4	13.2	5	0.0006	0.013	0.010
*Model 2:*							
Parental educational attainment	50.6	3	16.9	6.4	0.0003	0.012	0.010
*Model 3:*							
Family income	160.9	5	32.2	12.5	<0.0001	0.039	0.036
**Parent model**							
**Source of variation**	**Sum of square**	**df**	**Mean square**	**F value**	**Pr > F**	**Partial** **η**^**2**^	**Partial** **ω**^**2**^
*Covariates: patient characteristics*							
Patient sex	48.8	1	48.8	15.5	<0.0001	0.010	0.009
Patient race	21.8	1	21.8	6.9	0.0086	0.004	0.004
Patient age	0.5	1	0.5	0.2	0.6759	0.000	0.000
Current cardiac status of patient	291.3	3	94.1	30.9	<0.0001	0.059	0.057
Original cardiac diagnosis of patient	204.9	4	51.2	16.3	<0.0001	0.042	0.039
*Model 1:*							
Hollingshead index	89.3	4	22.3	7.1	<0.0001	0.018	0.016
*Model 2:*							
Parental educational attainment	112.6	3	37.5	12.0	<0.0001	0.023	0.021
*Model 3:*							
Family income	249	5	49.8	16.4	<0.0001	0.050	0.047

## Discussion

This study is one of the few to test multiple SES measures on a critical health-related outcome in the pediatric population and since SES measures for both patient and parent were based upon the parent’s responses to SES items, these can be considered valid measures of the family’s socio-economic status. Throughout the study, family income demonstrated the strongest fidelity in predicting HRQOL scores, while the impact of parental educational attainment level and the Hollingshead Index on HRQOL scores was somewhat muted. For the patient models, family income (η^2^ = 0.039) had 3 times higher the amount of variance explained compared to either the Hollingshead Index or parental educational attainment level. In the parent model, parental educational attainment level increased the η^2^ by 0.024 when controlling for covariates; however, family income by itself significantly increased the amount of variance explained by more than twice that amount, (η^2^ by 0.050). When examining the final model with parental educational attainment level and family income, since parental educational attainment level was not significant, while family income remained significantly related to parent-reported PCQLI score, we can conclude that family income by itself shows the strongest relationship with both patient- and parent-reported PCQLI Total score. While 4-5% of the variance explained by family income may seem small, the correlation coefficient associated with an η^2^ of that size is approximately 0.20, indicating a low moderate effect size [20]. Even though this effect may not have much clinical relevance for individual patients, for large populations, understanding the influence of family income could inform the level of risk or protection for health related quality of life based on SES, over and beyond health-related factors.

Our results regarding family income as a predictor of HRQOL were consistent with other studies that examined the relationship between income and health status. Even though this study did not translate family income into percent Federal Poverty Level (FPL), our findings were similar to the Newacheck’s [24] study on health care disparities among adolescents. They found strong gradient differentials between income (FPL) and general health status. Additionally, our results seem to confirm the existence of social gradients and aspects of health in adolescents found in Starfield’s study [25].

For many, family income is perceived as only associated with problems of health care access and utilization, and these problems are currently being addressed by programs such as Medicaid and the State Children’s Health Insurance Program (SCHIP). While these associations are true, other income-related issues are just as salient. For example, income levels are positively associated with better “nutrition, housing, schooling, and recreation,” [26] all elements relevant to an individual’s HRQOL. Individuals either living in poverty or near the poverty line are more likely to have problems with access to health care, have lower rates of health care utilization, and report that they have less satisfaction with care than individuals with higher SES scores [24,27,28]. Furthermore these SES factors have a deleterious effect in that individuals at the lower ends of the SES spectrum have higher rates of morbidity and mortality [29,30]. Fewer financial barriers to accessing health services might result in higher rates of preventative care and ultimately a healthier population.

Parental education as a proxy for SES, as suggested by Winkleby [31], did not yield as much predictive power on HRQOL as family income. As previously stated, while income and elements of HRQOL are associated, parental education is related to other aspects of a child’s health. For example parental education has been shown to affect child cognitive development [32] specifically but may only be broadly associated with HRQOL.

Finally, many researchers might be hesitant to ask income questions for their projects, relying on the Hollingshead Index instead. The Hollingshead Index requires two pieces of information: 1) parental educational attainment level and 2) occupation. On theoretical terms, the occupational component is much more problematic. Relying on a job title as an indicator of a person’s position within the social structure without any knowledge of their potential income may not be the best way to measure SES. Statistically, the Hollingshead Index had the lowest predictive power of the three indices. If researchers are going to take the time to ask sensitive questions about socio-economic status, results from this study indicate that income can be used in studies even if the categories have a broad bandwidth. These broader categories may allow respondents to be comfortable enough to answer the income question without feeling as though the question is intrusive. More importantly, however, is the need for researchers to re-examine the use of the Hollingshead Index as a measure of SES.

Further studies are needed to test the individual-level income categories against other measures of SES recently developed with the use of geocoding on HRQOL instruments, including the PCQLI. Although there have been significant limitations in using community-level geocodes for individual-level data [33,34], others have noted positive results from the use of geocodes [35,36]. If an SES measure can easily be developed with the use of geocodes and have as strong a predictive value as family income, researchers may have another SES tool with which to work. A second recommendation would be using, whenever possible, multiple measures of SES [15] Adler suggests using multiple determinants of SES to examine, and ultimately eliminate or reduce, disparities in health [26]. With parental education and family income having a moderate correlation, these two SES measures are capturing different aspects of socio-economic status. Parental education is a simple variable to collect, and as shown in this study, most participants are willing to provide family income information if the income categories are broad enough.

### Limitations

This study only had a single time point of analysis and thus cannot show change over time. Additionally the income distribution for the sample does not reflect the general population of the United States. Other limitations include the potential for bias due to the exclusionary criterion of English-speaking only.

## Conclusion

Family income as an SES measure had the greatest influence on PCQLI Total score across respondent groups and explained more of the variation compared to the Hollingshead Index or highest parental educational attainment level. Having a clear idea of the relationship between socio-economic status indicators and HRQOL will enable researchers to make an unbiased assessment of the results of studies assessing HRQOL and thereby develop more effective future interventions to improve HRQOL.

## Competing interest

The authors declare that they have no competing interests.

## Authors’ contribution

AEC conceived of the study, designed the study, performed the statistical analysis and drafted the manuscript. DD provided expertise on health-related quality of life studies as well as socio-economic status and its relationship to health disparities, and drafting the manuscript. RI provided guidance with statistical analysis and drafting the manuscript. SH edited the manuscript. GW, JWN, LM, KM, and MIC provided expertise on pediatric cardiology issues as well study coordination for each site. JW provided expertise on health-related quality of life and psychological issues within the larger study. BSM participated in the design of the study, provided expertise on pediatric cardiology and health-related quality of life, and helped draft the manuscript. All authors read and approved the final manuscript.
